# Supplementary material for: PRMT1 oligomerization regulates RNA-binding protein cascade to promote pancreatic cancer
Source: Life Sci Alliance. 2025 Jul 17;8(9):e202503202. doi: 10.26508/lsa.202503202 (PMC12272085; doi:10.26508/lsa.202503202)

Uncropped gel and blot images for main figures

Fig. 4J

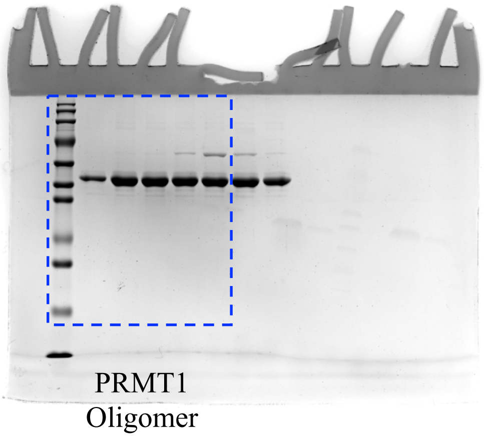

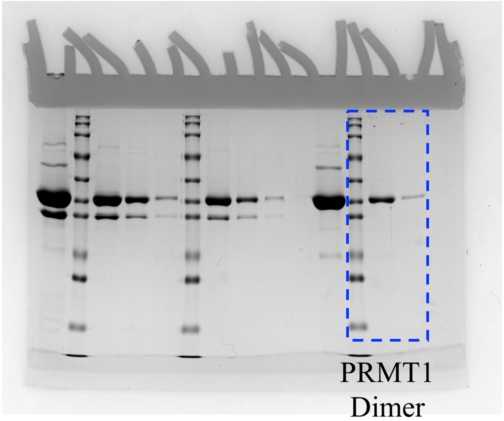

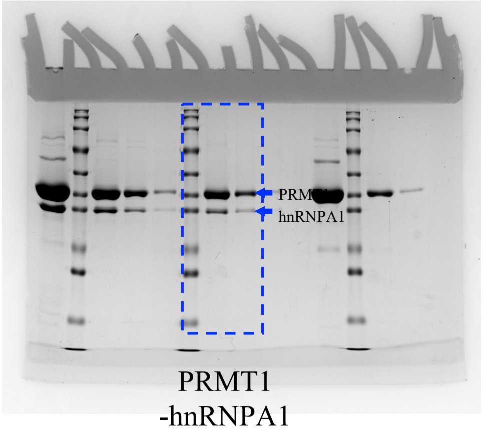

Fig. 5A.

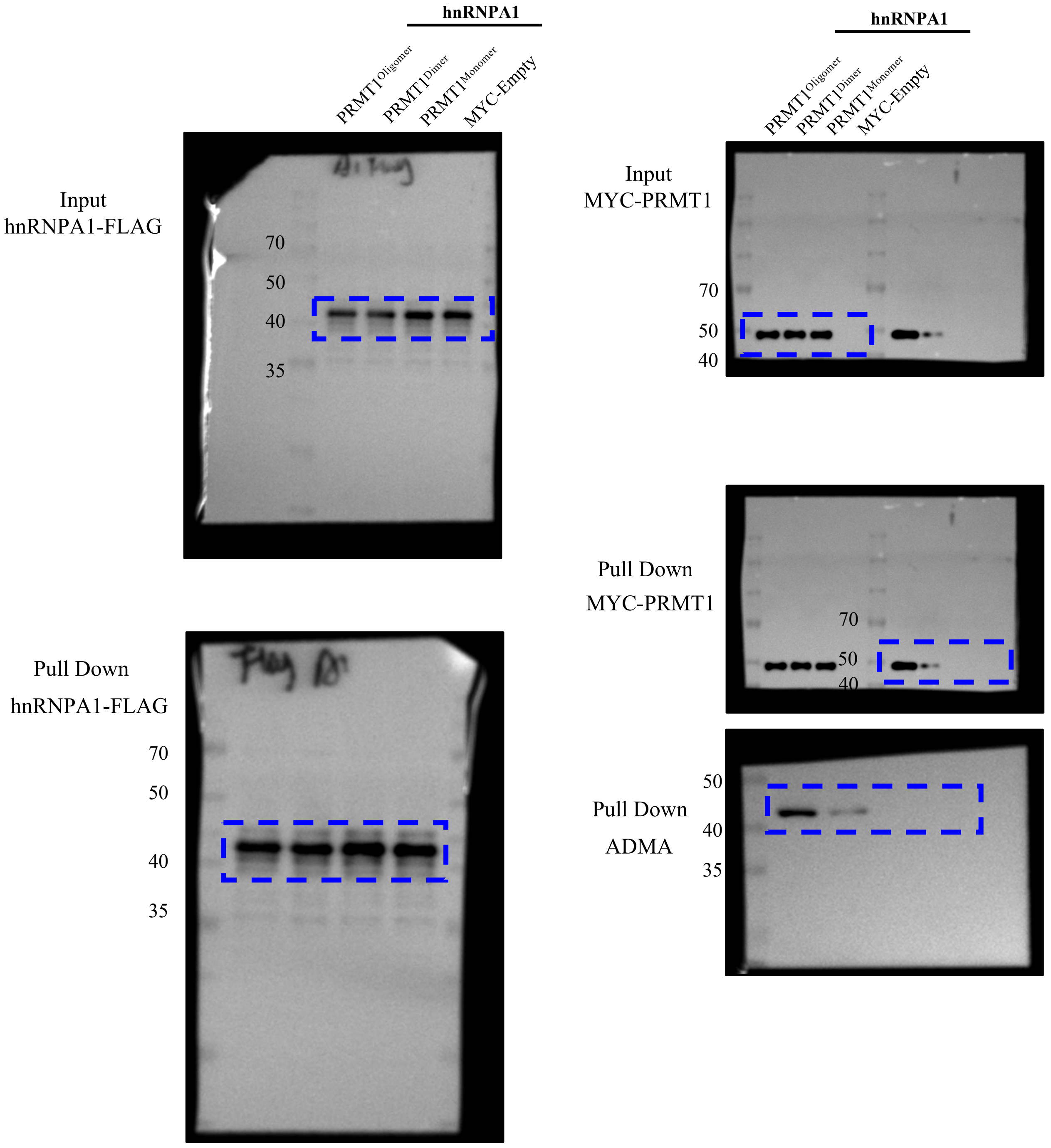
hnRNPA1
PRMT1Oligomer
PRMT1Dimer
PRMT1Monomer
MYC-Empty
Input
hnRNPA1-FLAG
70
50
40
35
Pull Down
hnRNPA1-FLAG
70
50
40
35
hnRNPA1
PRMT1Oligomer
PRMT1Dimer
PRMT1Monomer
MYC-Empty
Input
MYC-PRMT1
70
50
40
Pull Down
MYC-PRMT1
70
50
40
Pull Down
ADMA
50
40
35

Fig.5B

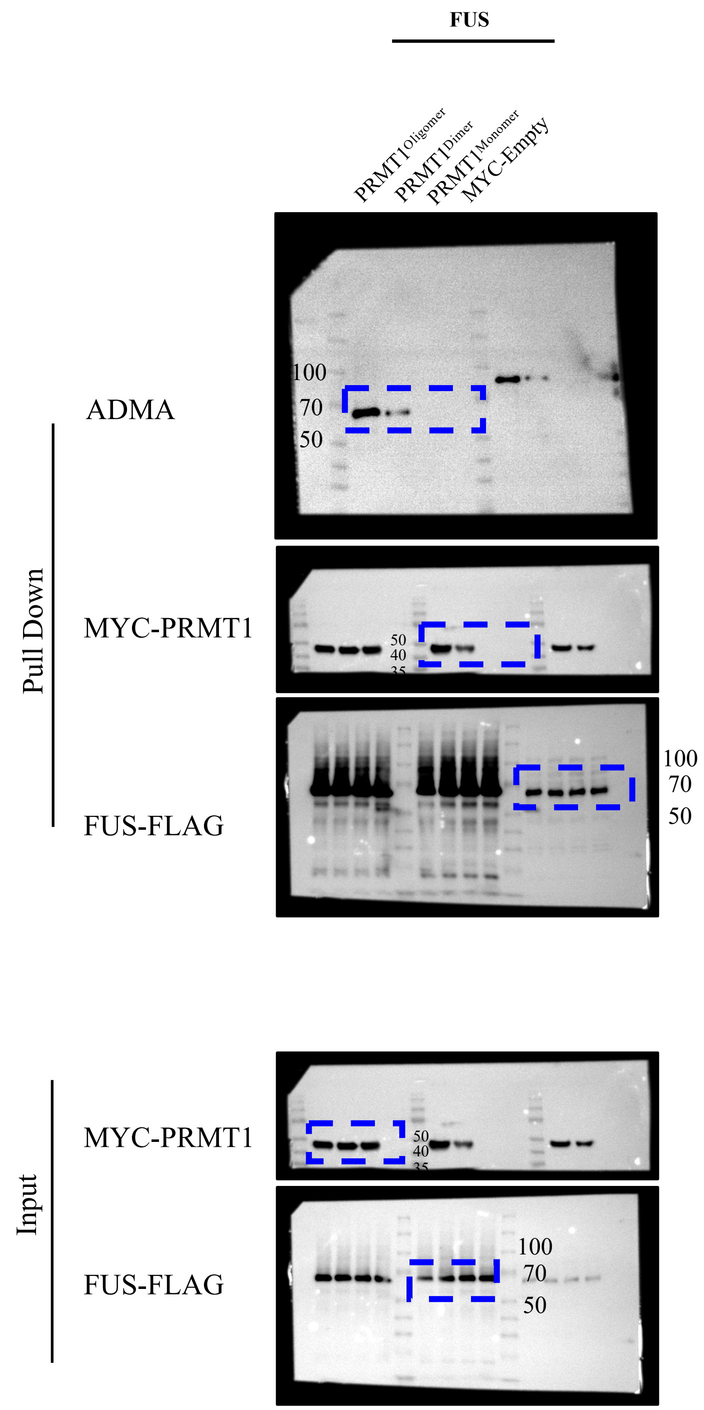

FUS
PRMT1Oligomer
PRMT1Dimer
PRMT1Monomer
MYC-Empty
ADMA
100
70
50
Pull Down
MYC-PRMT1
50
40
FUS-FLAG
100
70
50
Input
MYC-PRMT1
50
40
FUS-FLAG
100
70
50

Fig.5C

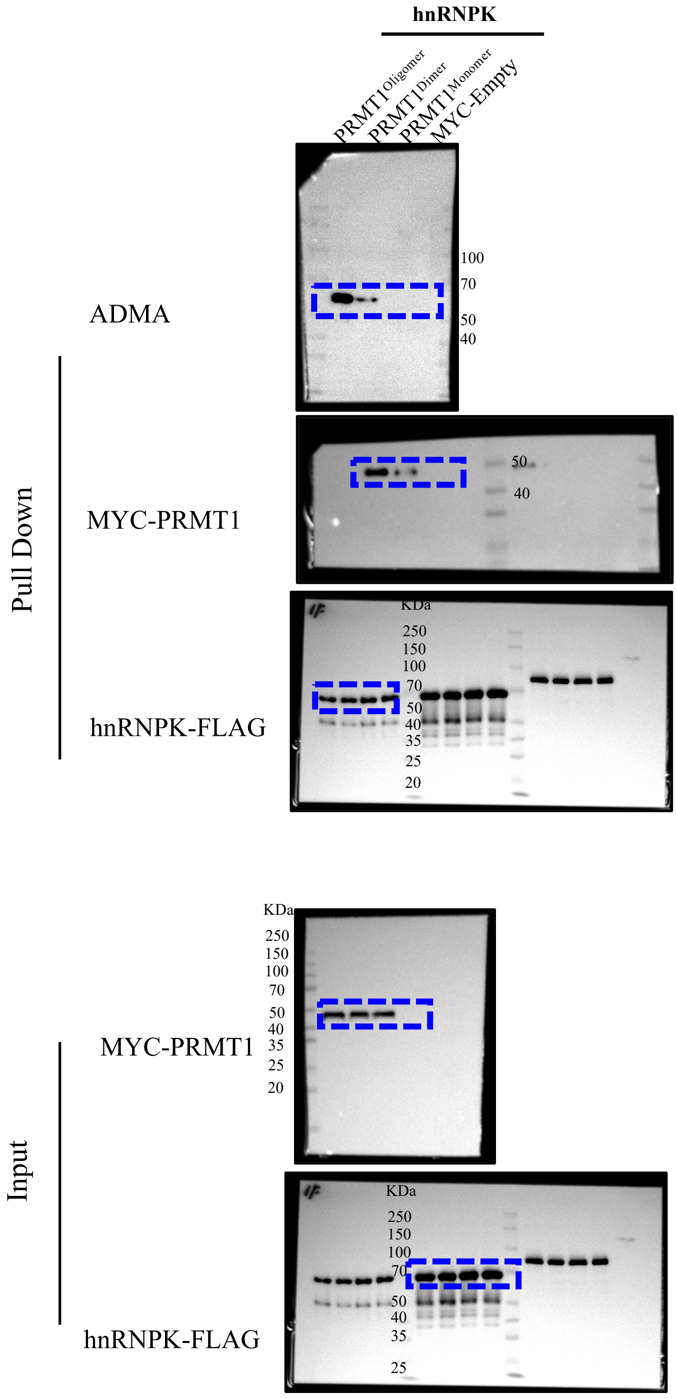
hnRNPK
PRMT1Oligomer
PRMT1Dimer
PRMT1Monomer
MYC-Empty
ADMA
100
70
50
40
Pull Down
MYC-PRMT1
50
40
hnRNPK-FLAG
KDa
250
150
100
70
50
40
35
25
20
Input
MYC-PRMT1
KDa
250
150
100
70
50
40
35
25
20
hnRNPK-FLAG
KDa
250
150
100
70
50
40
35
25

Fig.5D

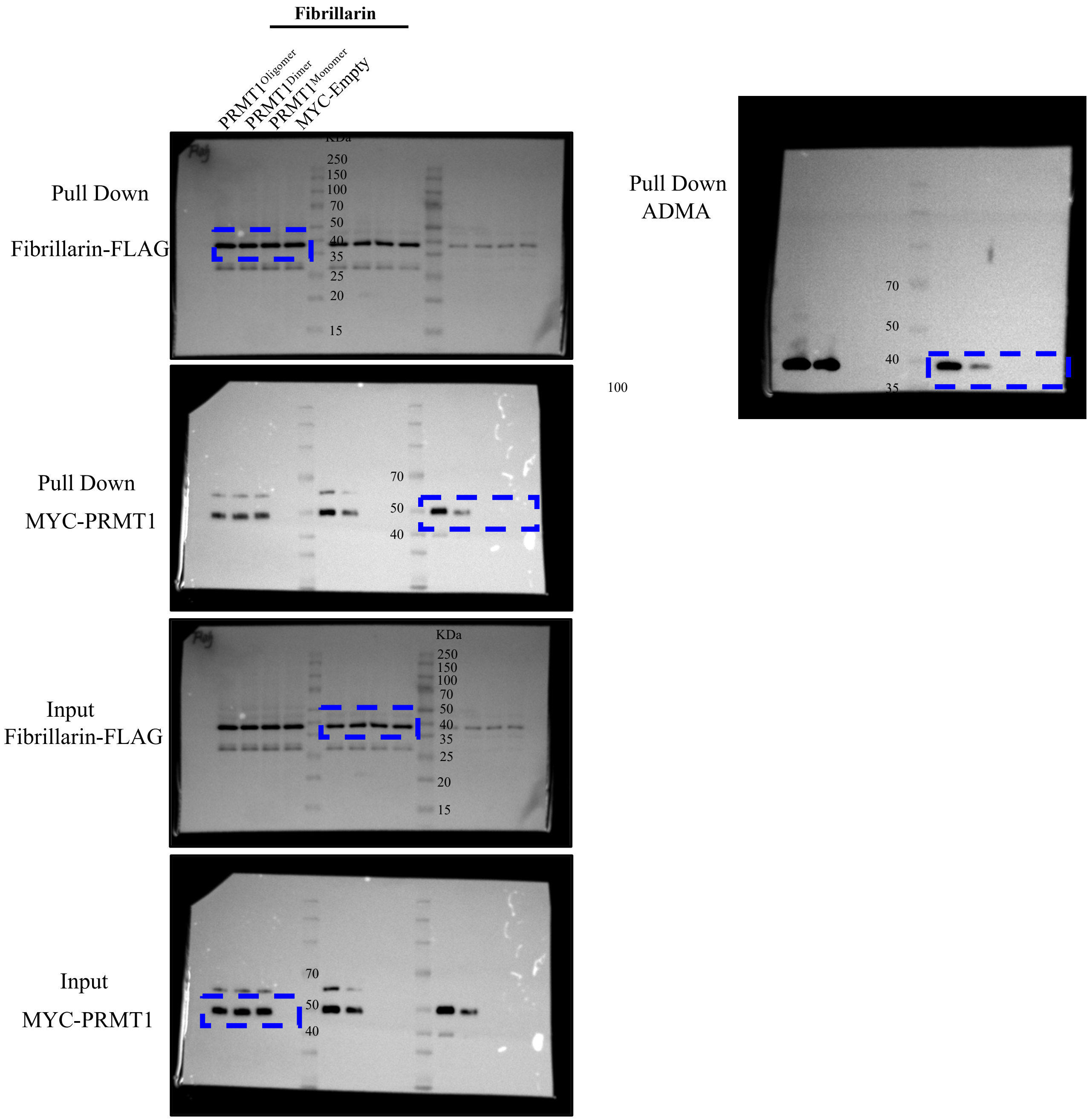
Fibrillarin
PRMT1 Oligomer
PRMT1 Dimer
PRMT1 Monomer
MYC-Empty
Pull Down
Fibrillarin-FLAG
KDa
250
150
100
70
50
40
35
25
20
15
Pull Down
MYC-PRMT1
70
50
40
Input
Fibrillarin-FLAG
KDa
250
150
100
70
50
40
35
25
20
15
Input
MYC-PRMT1
70
50
40
100
Pull Down
ADMA
70
50
40
35

Fig. 5E

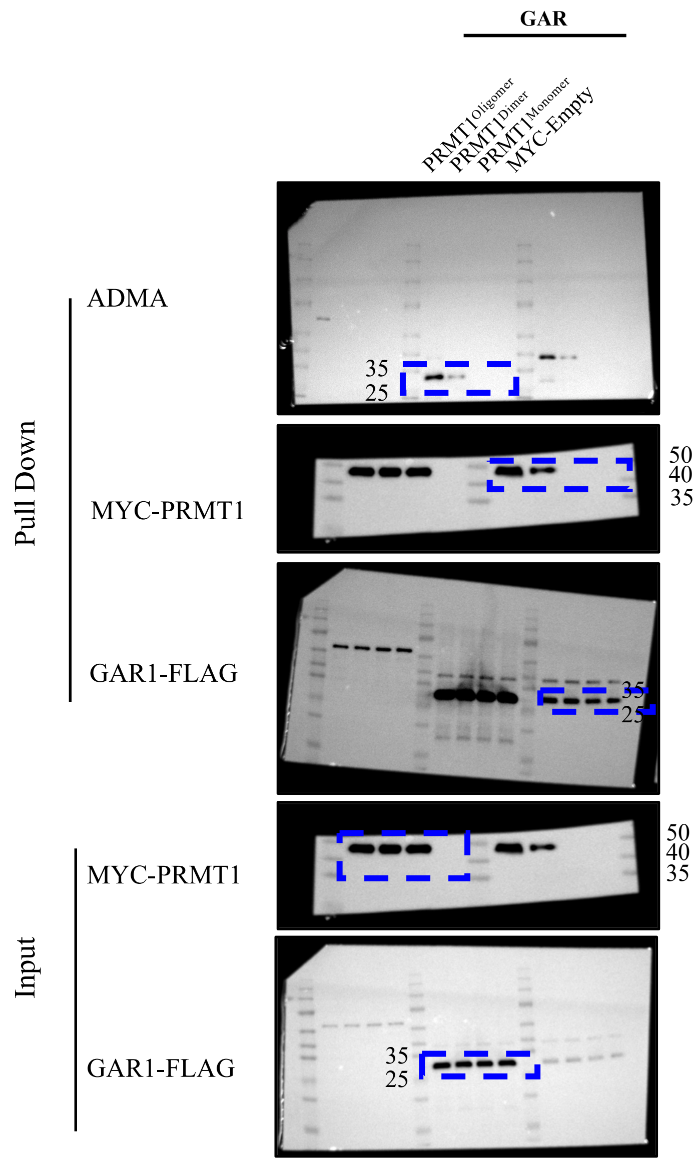
GAR
PRMT1Oligomer
PRMT1Dimer
PRMT1Monomer
MYC-Empty
Pull Down
ADMA
35
25
MYC-PRMT1
50
40
35
GAR1-FLAG
35
25
Input
MYC-PRMT1
50
40
35
GAR1-FLAG
35
25

Fig.5F

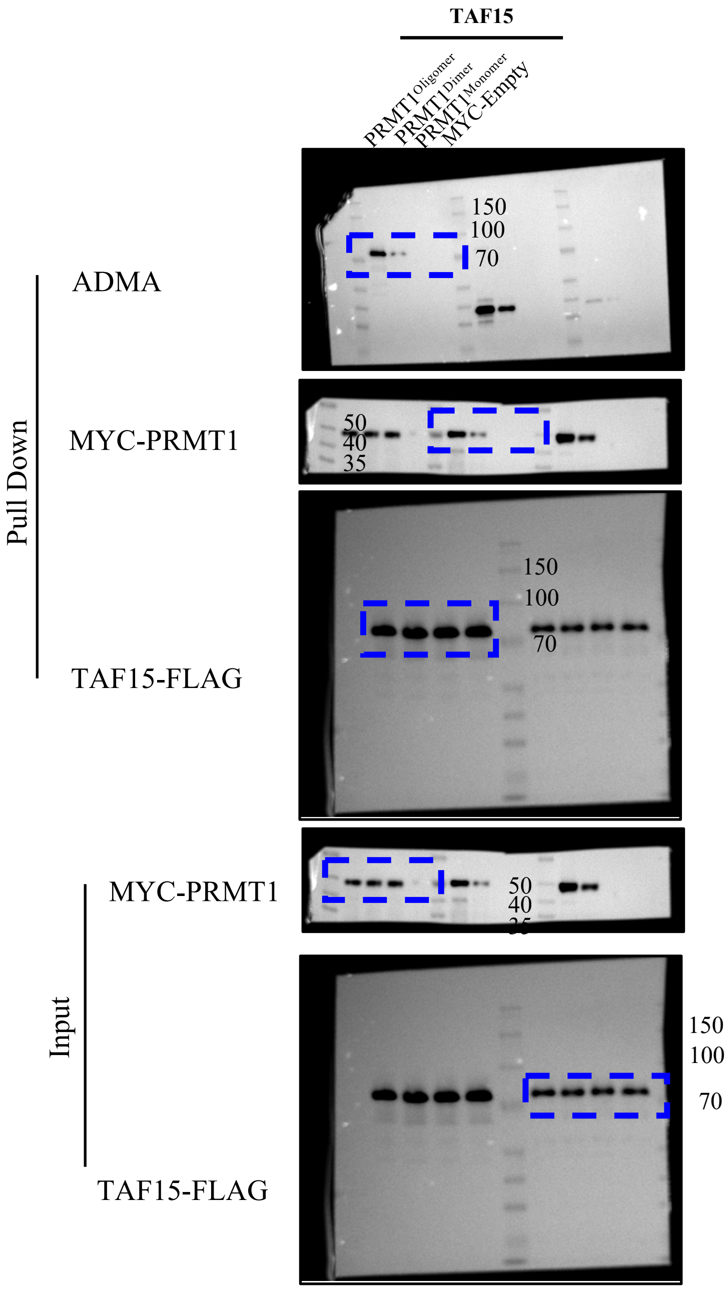
TAF15
PRMT1Oligomer
PRMT1Dimer
PRMT1Monomer
MYC-Empty
Pull Down
ADMA
150
100
70
MYC-PRMT1
50
40
35
TAF15-FLAG
150
100
70
Input
MYC-PRMT1
50
40
TAF15-FLAG
150
100
70

Fig.5G

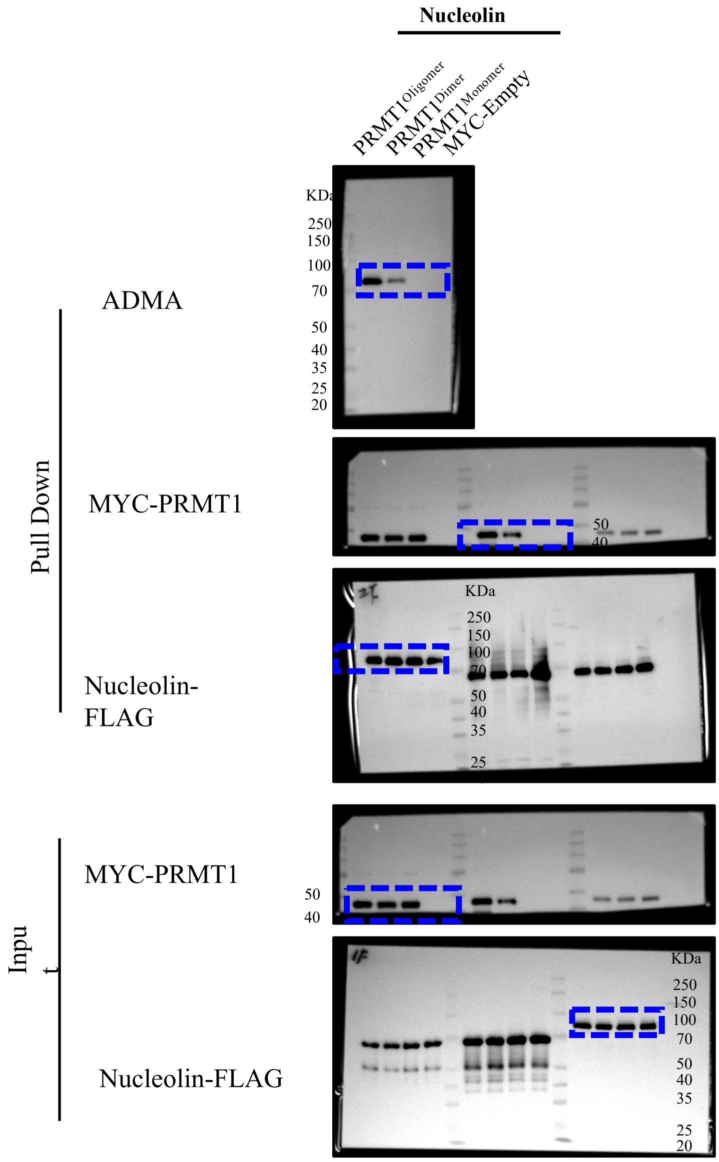

Nucleolin
PRMT1 Oligomer
PRMT1 Dimer
PRMT1 Monomer
MYC-Empty
KDa
250
150
100
70
50
40
35
25
20
ADMA
Pull Down
MYC-PRMT1
Nucleolin-FLAG
Input
MYC-PRMT1
Nucleolin-FLAG

Fig.5H

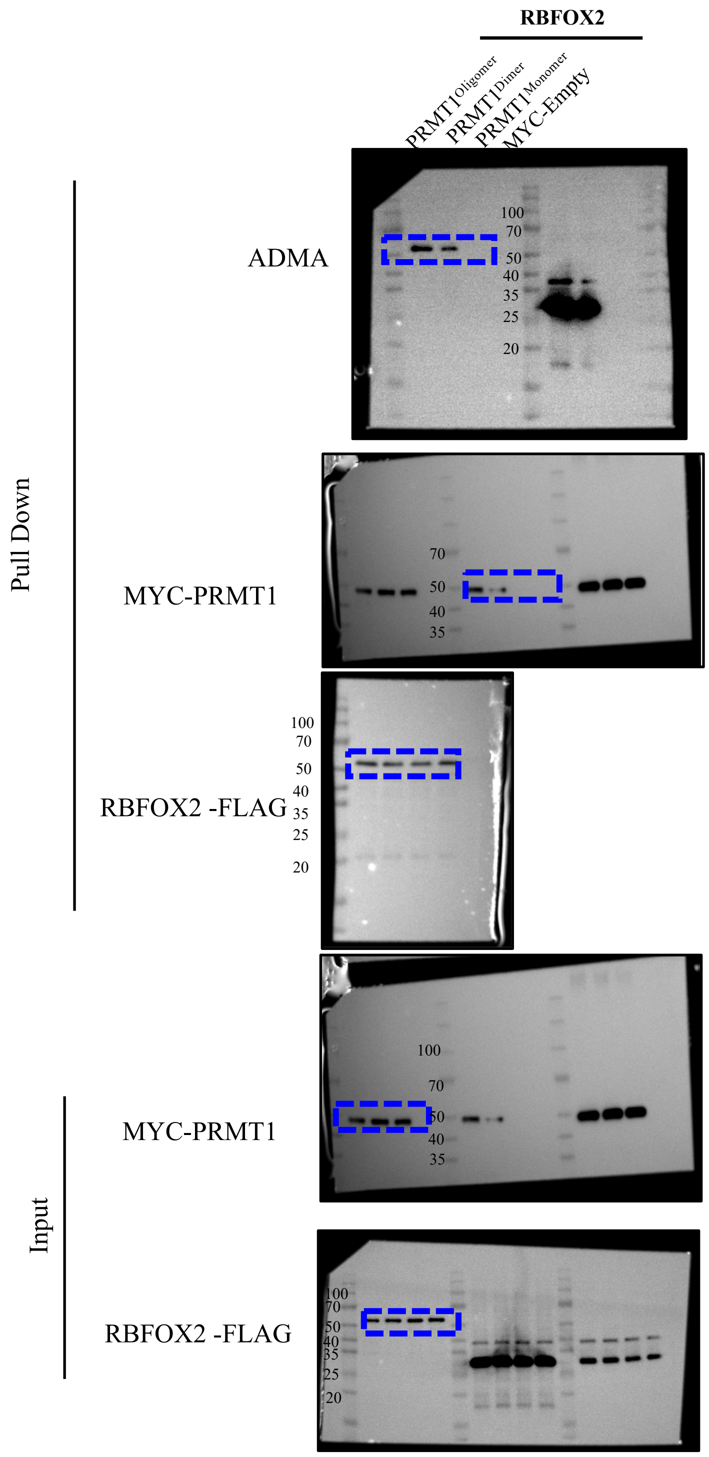
RBFOX2
PRMT1Oligomer
PRMT1Dimer
PRMT1Monomer
MYC-Empty
Pull Down
ADMA
100
70
50
40
35
25
20
MYC-PRMT1
70
50
40
35
RBFOX2 -FLAG
100
70
50
40
35
25
20
Input
MYC-PRMT1
100
70
50
40
35
RBFOX2 -FLAG
100
70
50
40
35
25
20

Fig.6A

PRTM1

GAPDH

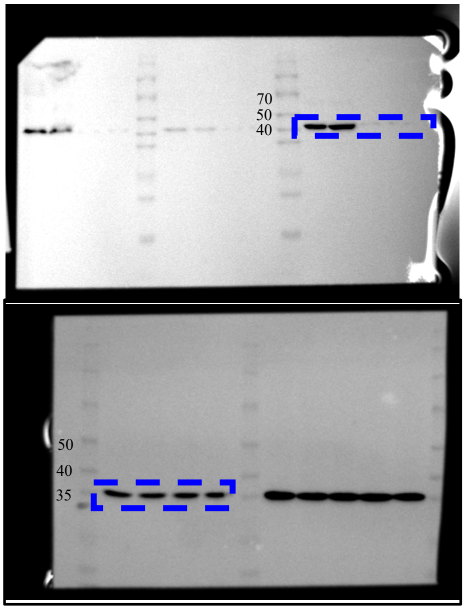

Fig.6B

PRTM1

GAPDH

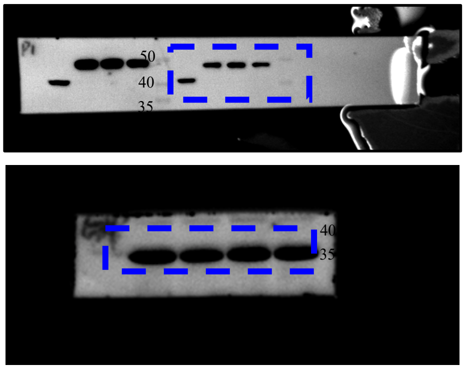

Fig. 6C

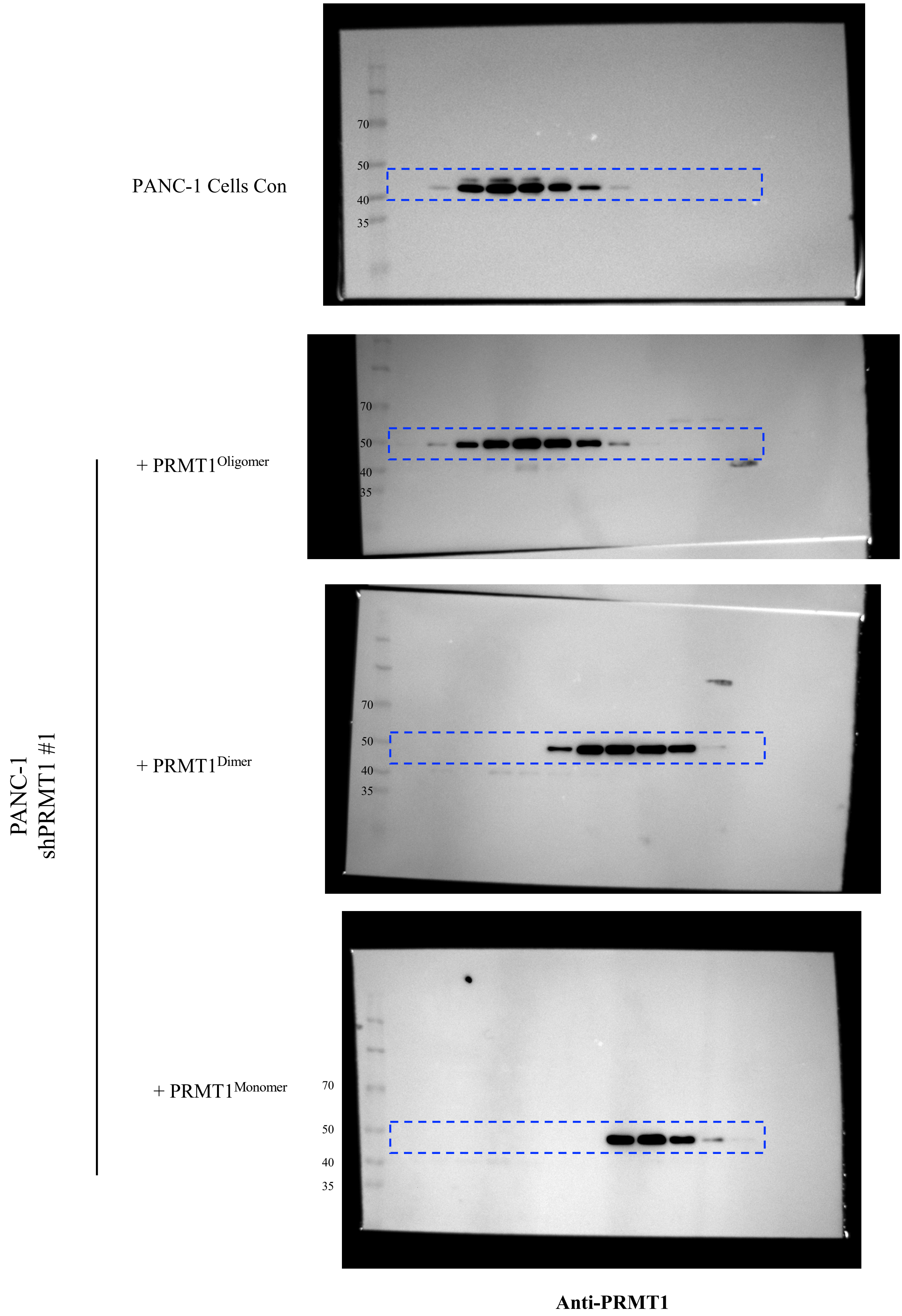
PANC-1 Cells Con
70
50
40
35
+ PRMT1 Oligomer
70
50
40
35
+ PRMT1 Dimer
70
50
40
35
+ PRMT1 Monomer
70
50
40
35
PANC-1
shPRMT1 #1
Anti-PRMT1

Fig. 6D

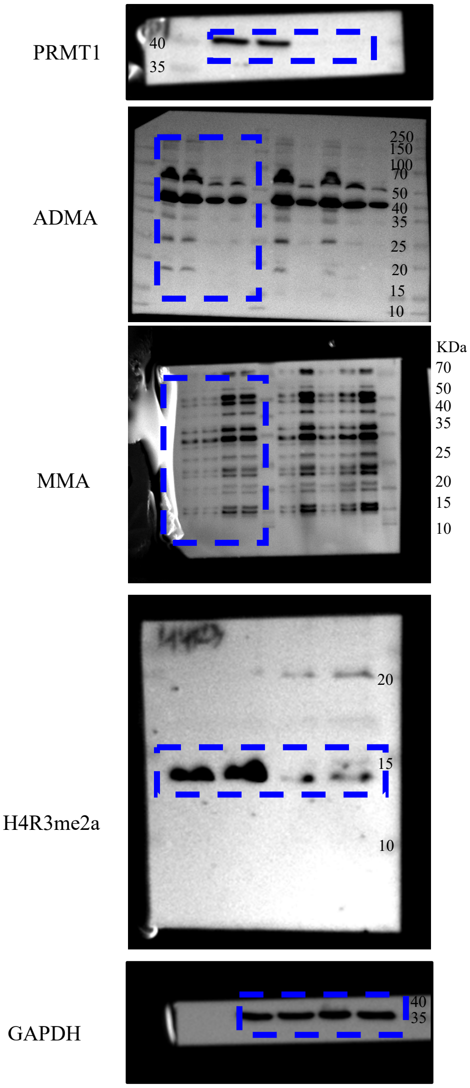

PRMT1
40
35
ADMA
250
150
100
70
50
40
35
25
20
15
10
MMA
KDa
70
50
40
35
25
20
15
10
H4R3me2a
20
15
10
GAPDH
40
35

Fig.6E

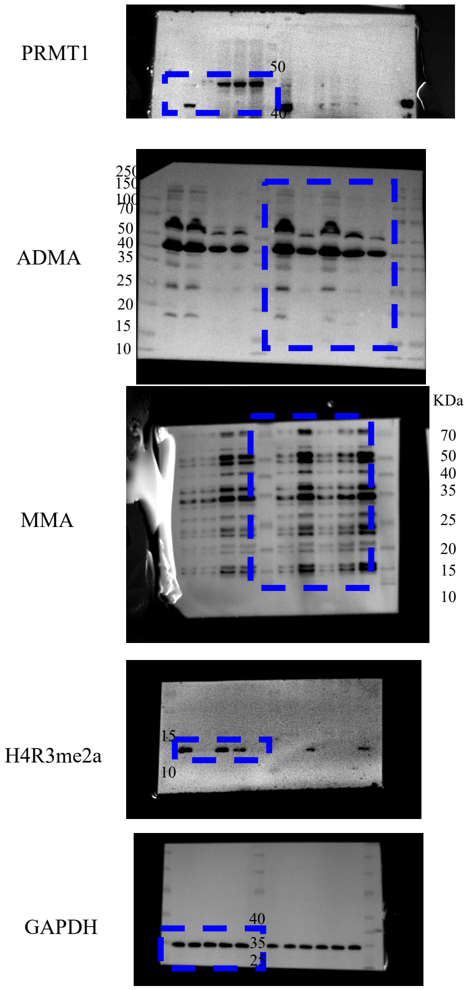

PRMT1
50
40
ADMA
250
150
100
70
50
40
35
25
20
15
10
MMA
KDa
70
50
40
35
25
20
15
10
H4R3me2a
15
10
GAPDH
40
35
25

Uncropped gel and blot images for Supplementary Figures

Fig. S1A

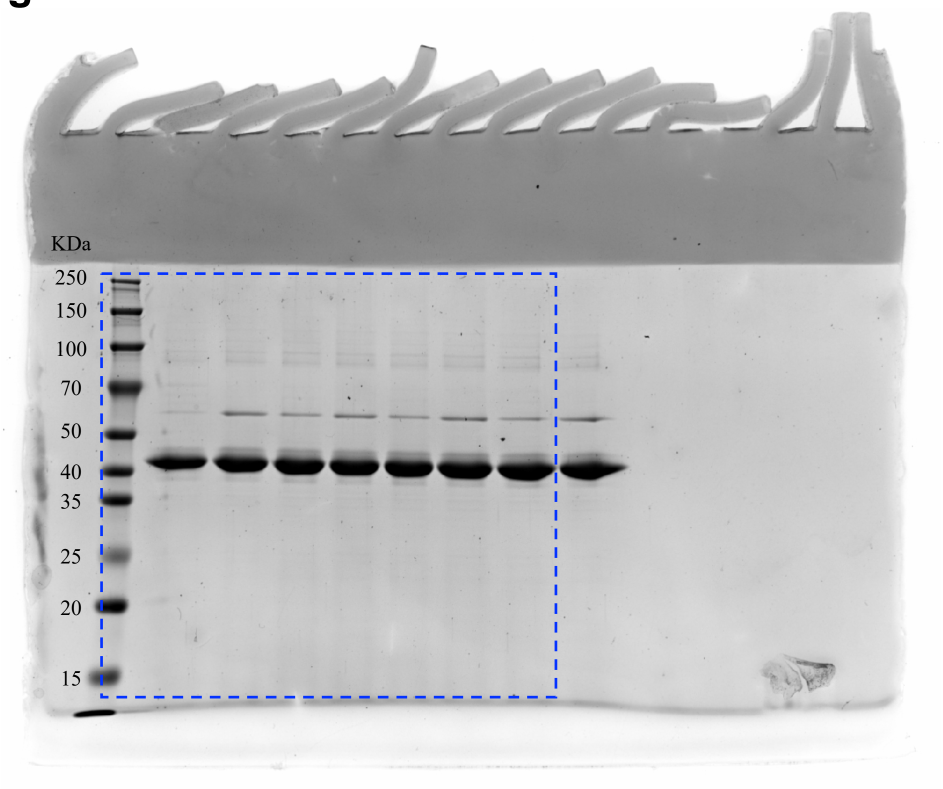

Fig.S3A

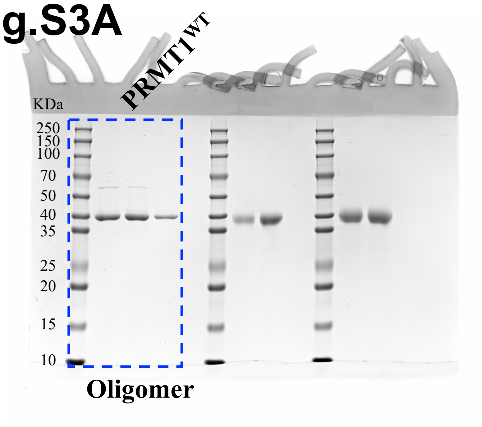

Fig.S3A

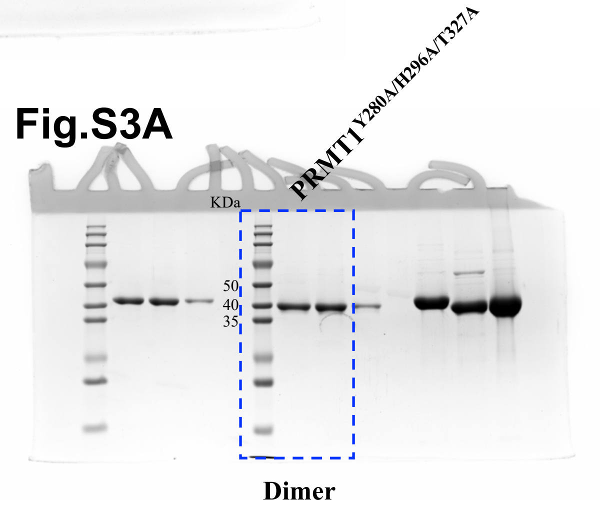

Fig.S3A

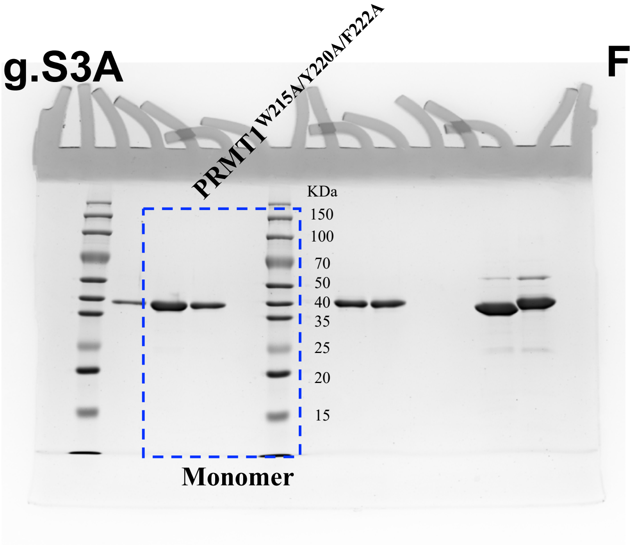

Fig. S3B

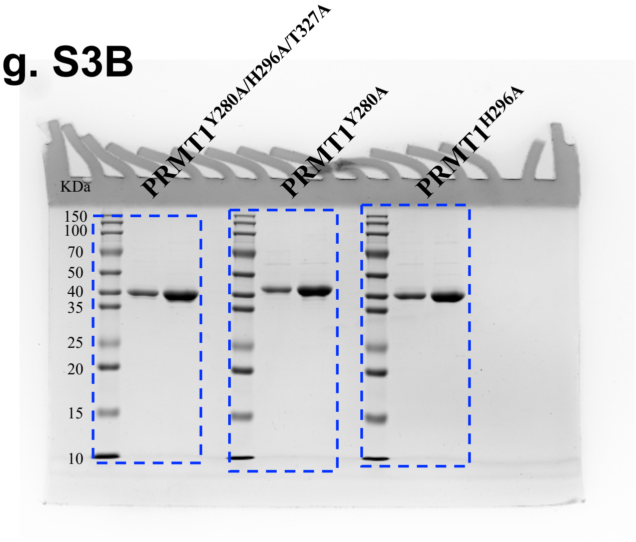

Fig.S3E

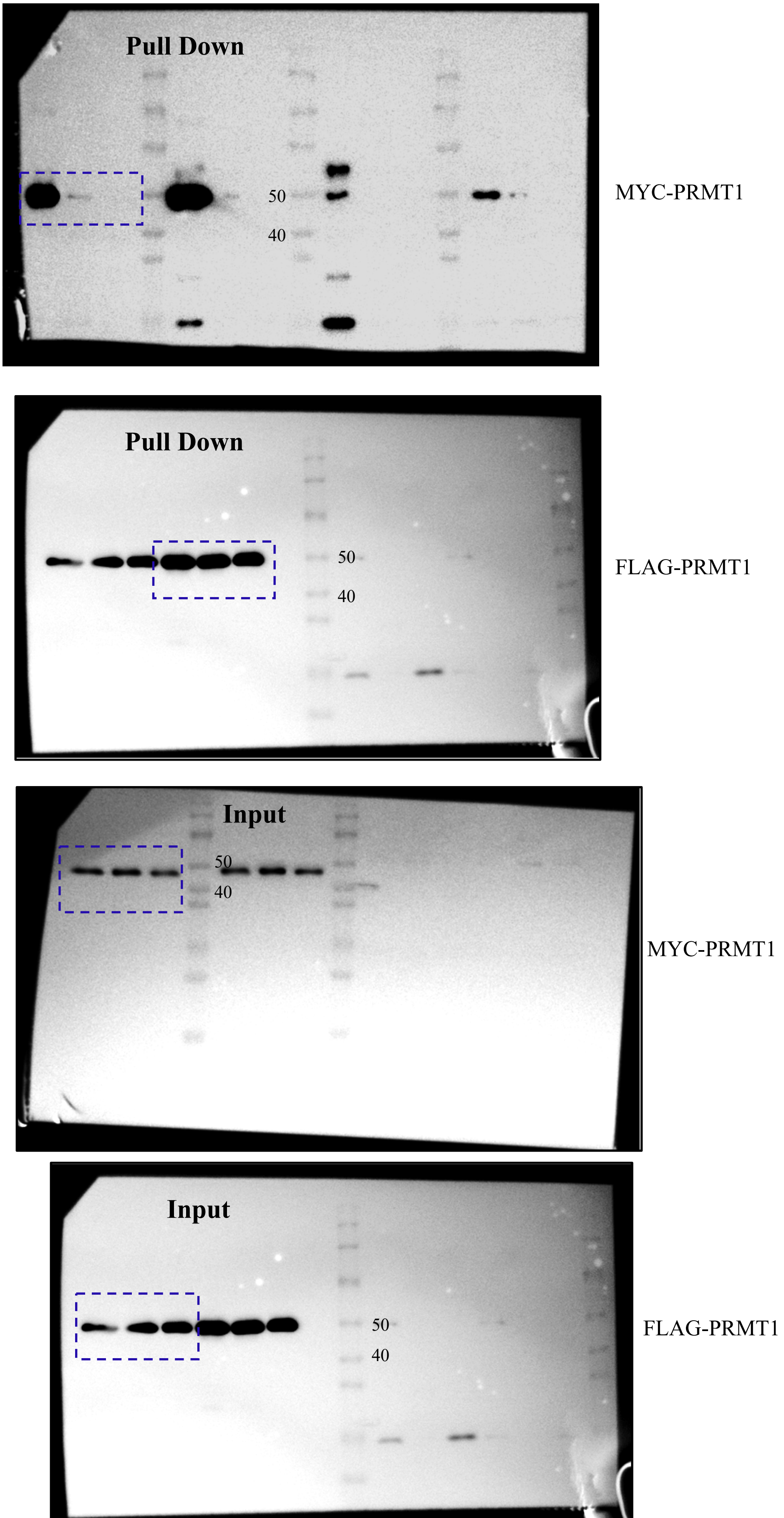

Pull Down
50
40
MYC-PRMT1
Pull Down
50
40
FLAG-PRMT1
Input
50
40
MYC-PRMT1
Input
50
40
FLAG-PRMT1

Fig.S3F

Pull Down

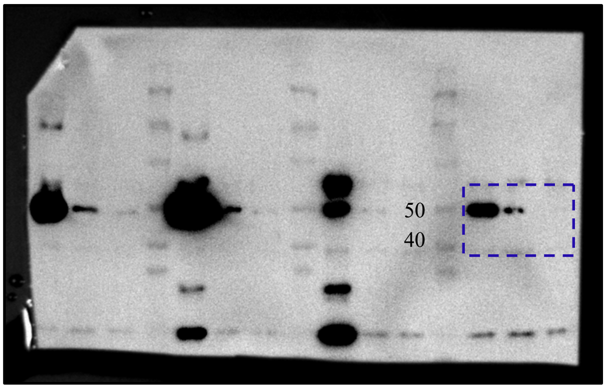

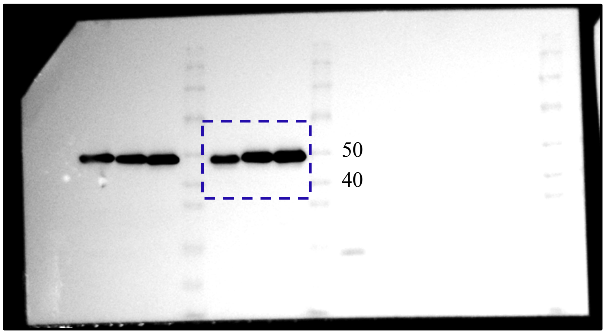

Input

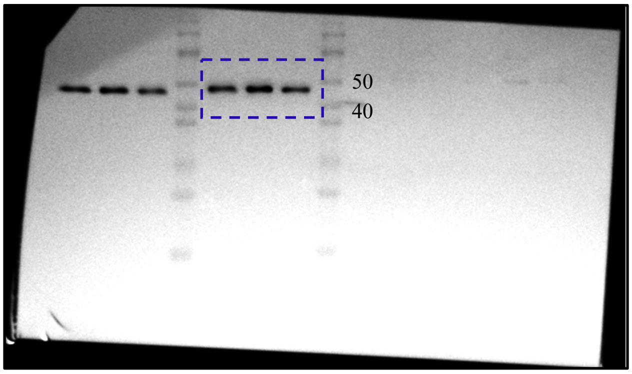

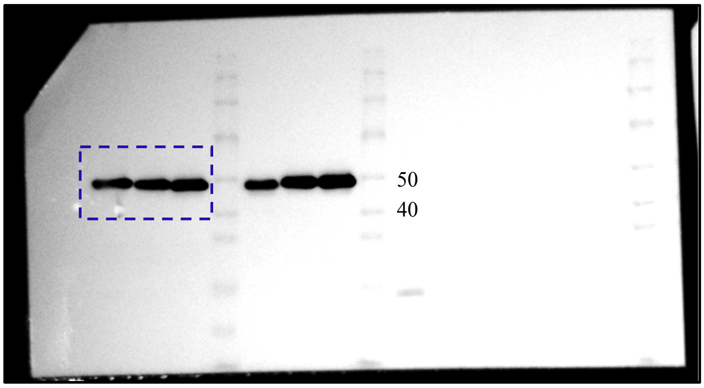

Fig.S4B

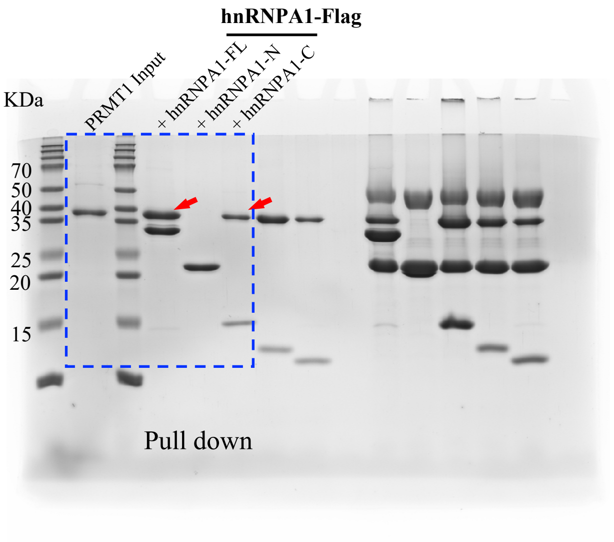

Fig.S4D

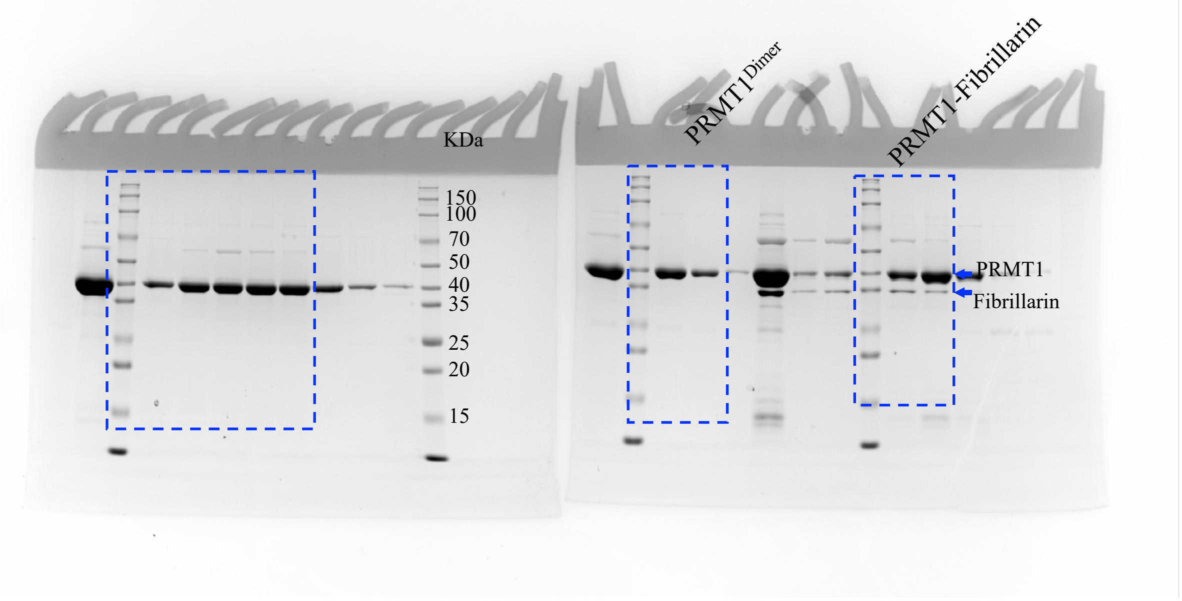

Fig.S5B

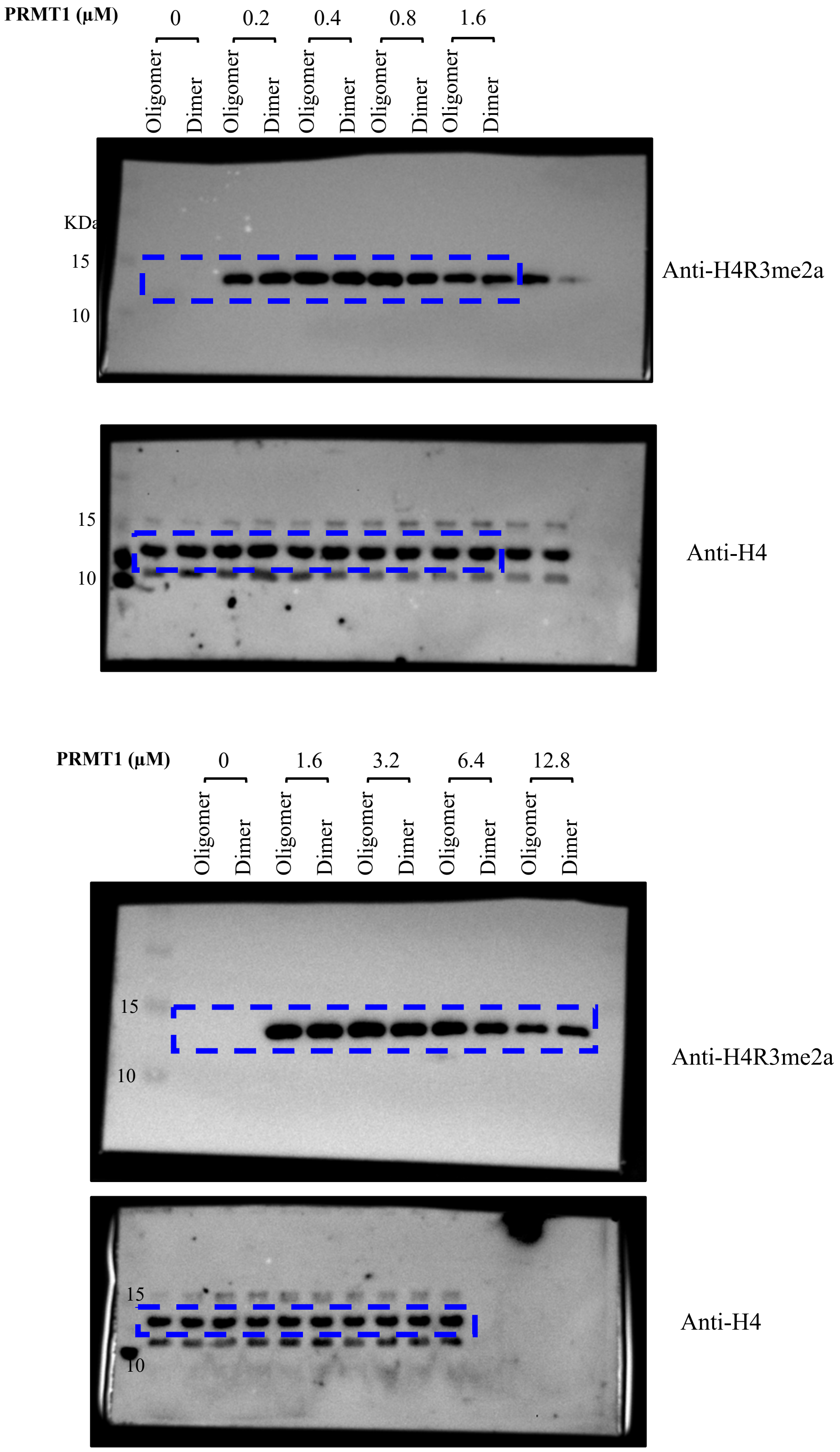
PRMT1 (μM)
0
0.2
0.4
0.8
1.6
Oligomer
Dimer
Oligomer
Dimer
Oligomer
Dimer
Oligomer
Dimer
Oligomer
Dimer
KDa
15
10
Anti-H4R3me2a
15
10
Anti-H4
PRMT1 (μM)
0
1.6
3.2
6.4
12.8
Oligomer
Dimer
Oligomer
Dimer
Oligomer
Dimer
Oligomer
Dimer
Oligomer
Dimer
15
10
Anti-H4R3me2a
15
10
Anti-H4

Fig.S5C.

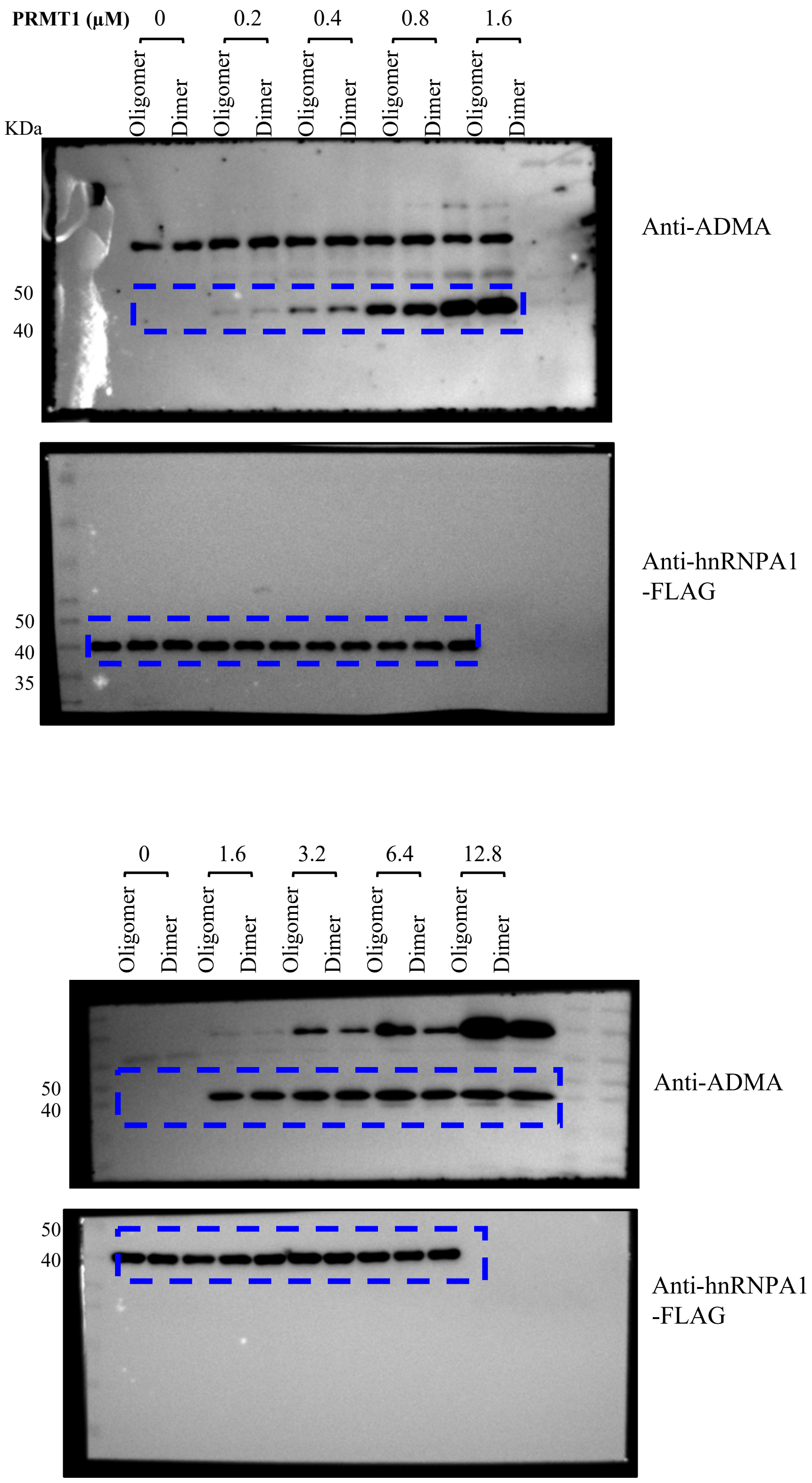
PRMT1 (μM)
0
0.2
0.4
0.8
1.6
Oligomer
Dimer
KDa
50
40
Anti-ADMA
50
40
35
Anti-hnRNPA1
-FLAG
0
1.6
3.2
6.4
12.8
Oligomer
Dimer
50
40
Anti-ADMA
50
40
Anti-hnRNPA1
-FLAG

Fig. S5D

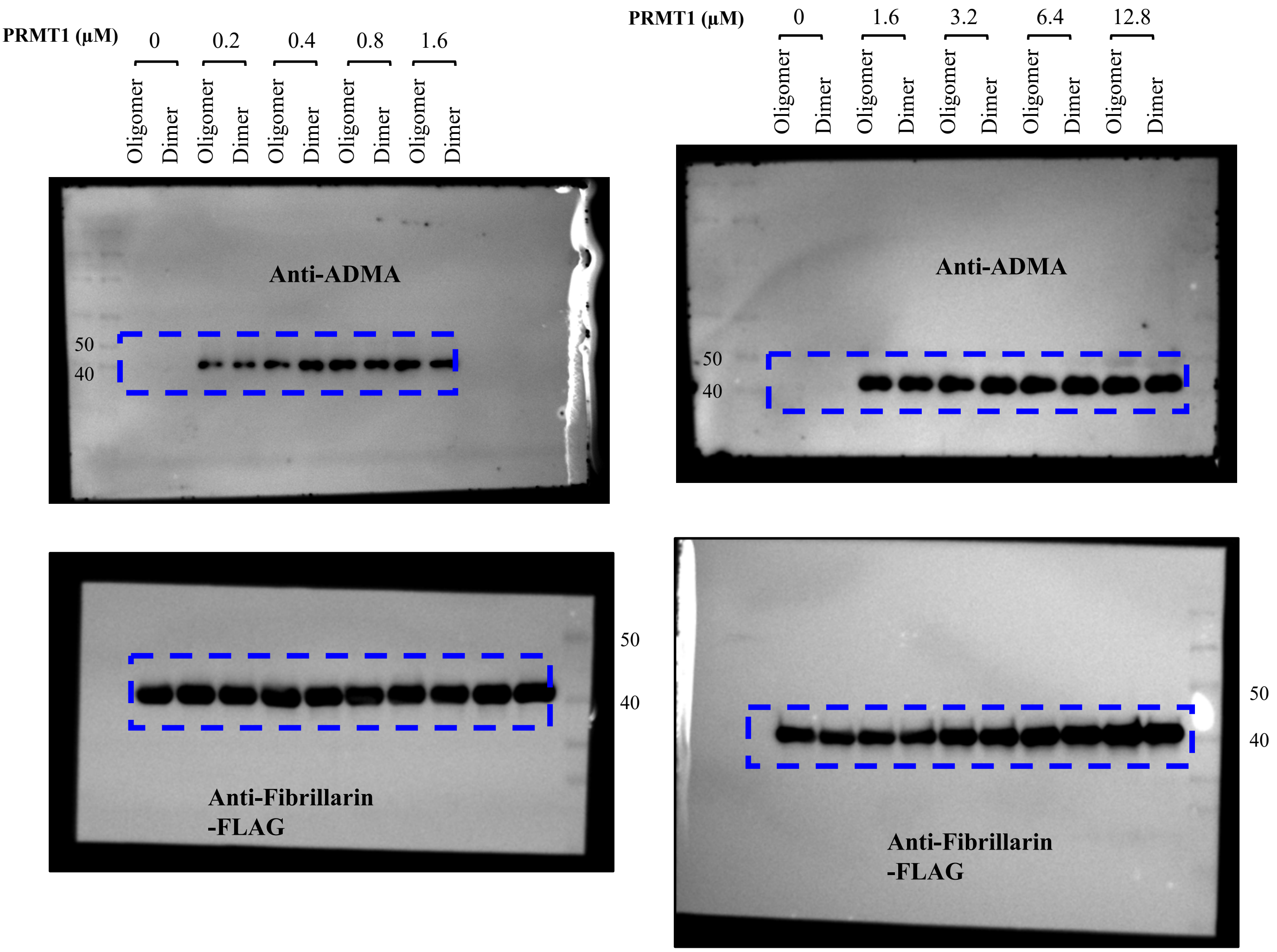
PRMT1 (µM)
0
0.2
0.4
0.8
1.6
Oligomer
Dimer
Anti-ADMA
50
40
Anti-Fibrillarin
-FLAG
PRMT1 (µM)
0
1.6
3.2
6.4
12.8
Oligomer
Dimer
Anti-ADMA
50
40
Anti-Fibrillarin
-FLAG

Fig.S6A

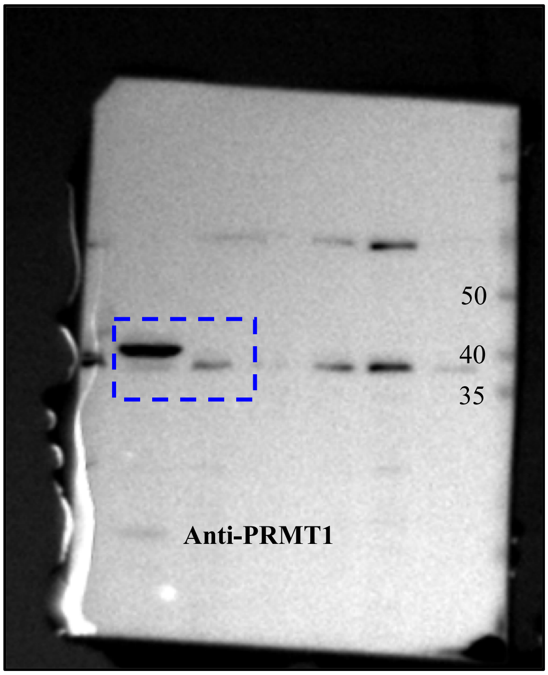

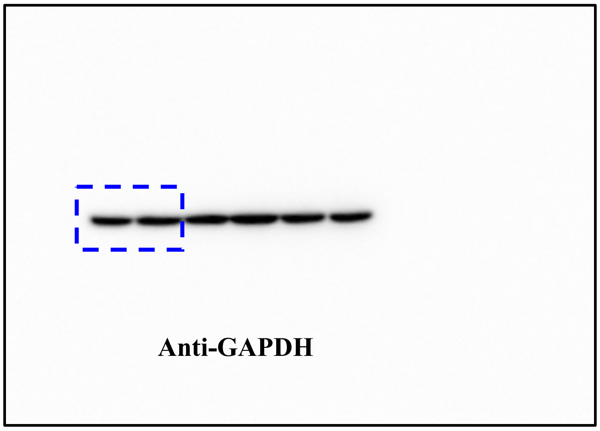

Fig.S6C.

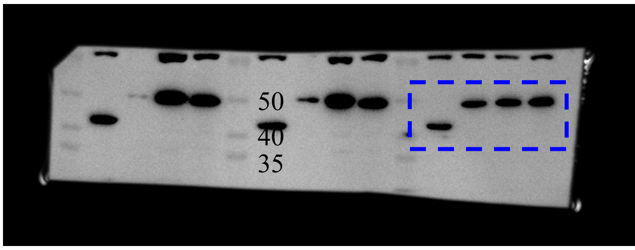

Anti-PRMT1

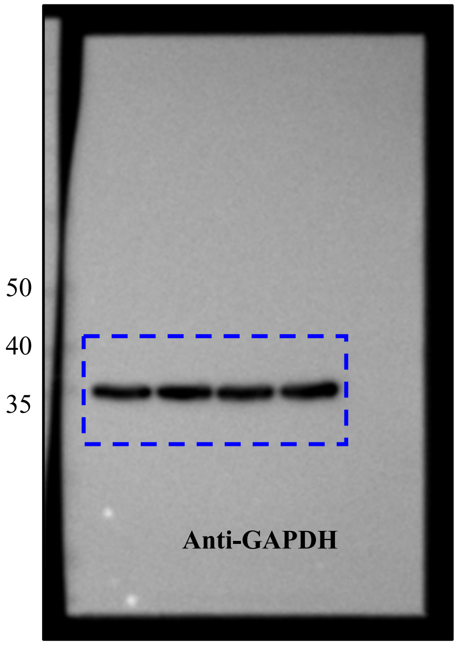

Fig.S6D

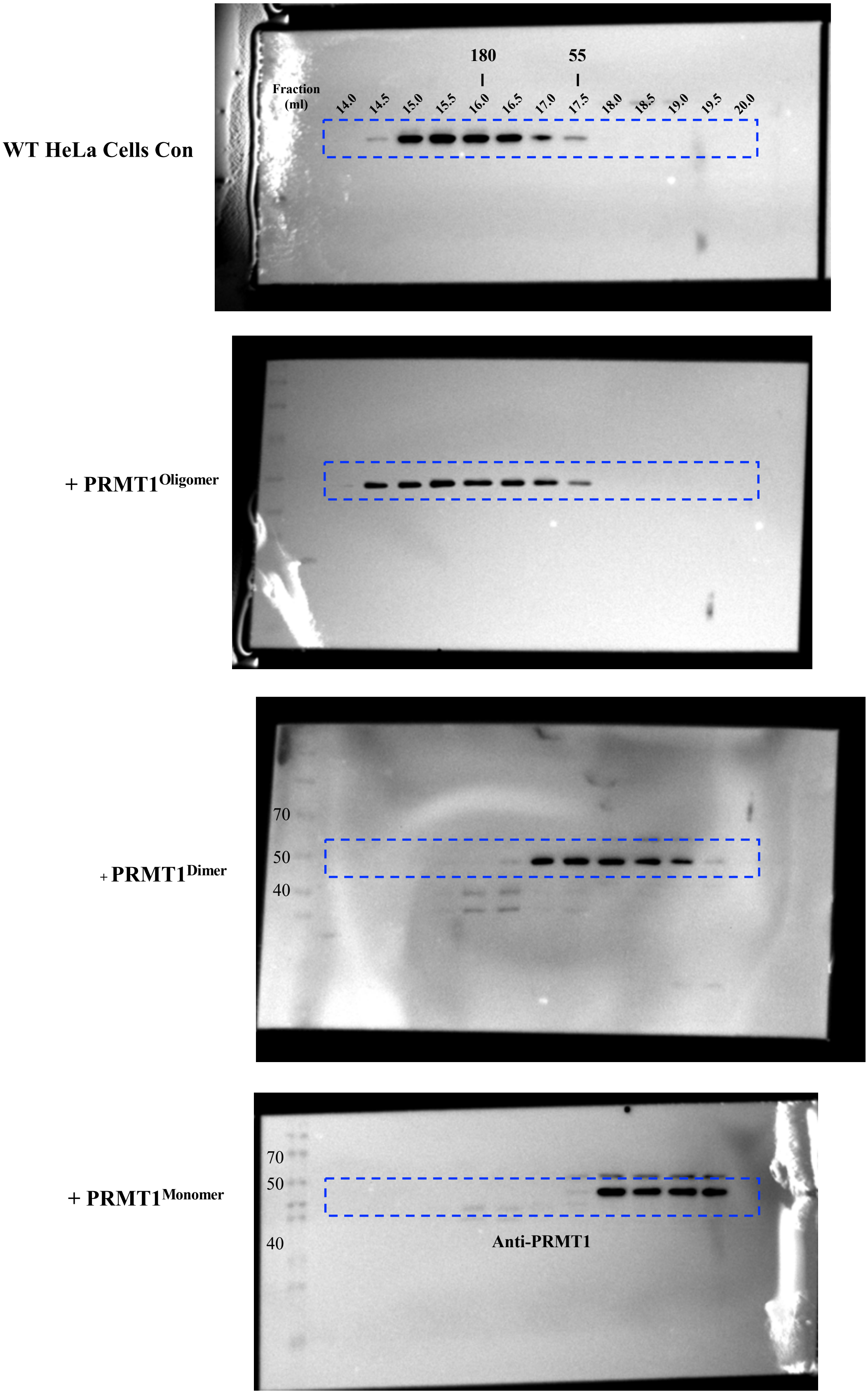
WT HeLa Cells Con
Fraction (ml)
180
55
14.0
14.5
15.0
15.5
16.0
16.5
17.0
17.5
18.0
18.5
19.0
19.5
20.0
+ PRMT1Oligomer
+PRMT1Dimer
70
50
40
+ PRMT1Monomer
70
50
40
Anti-PRMT1

Fig.S6E

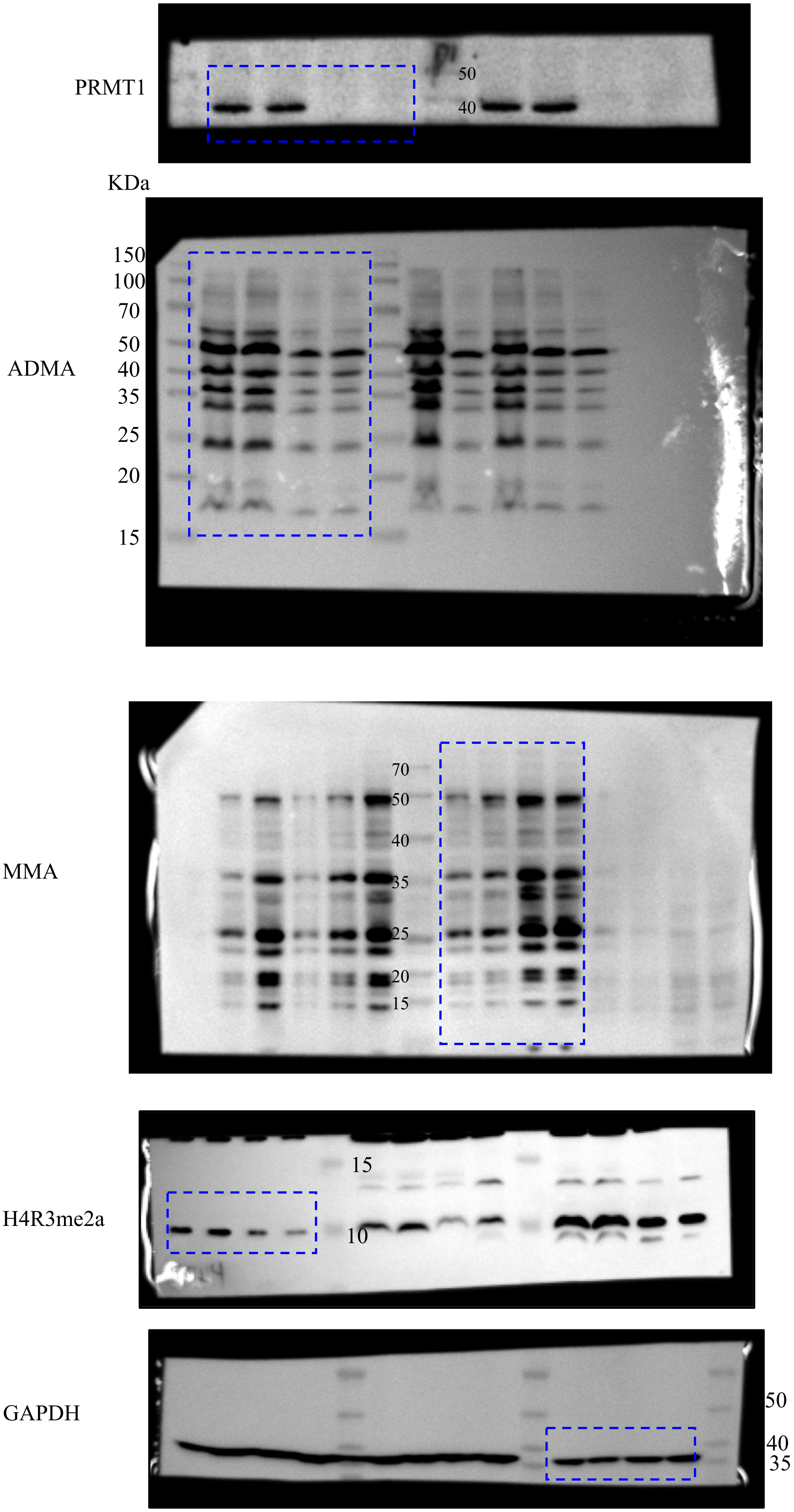
PRMT1
50
40
KDa
150
100
70
50
40
35
25
20
15
ADMA
MMA
70
50
40
35
25
20
15
H4R3me2a
15
10
GAPDH
50
40
35

Fig.S7A

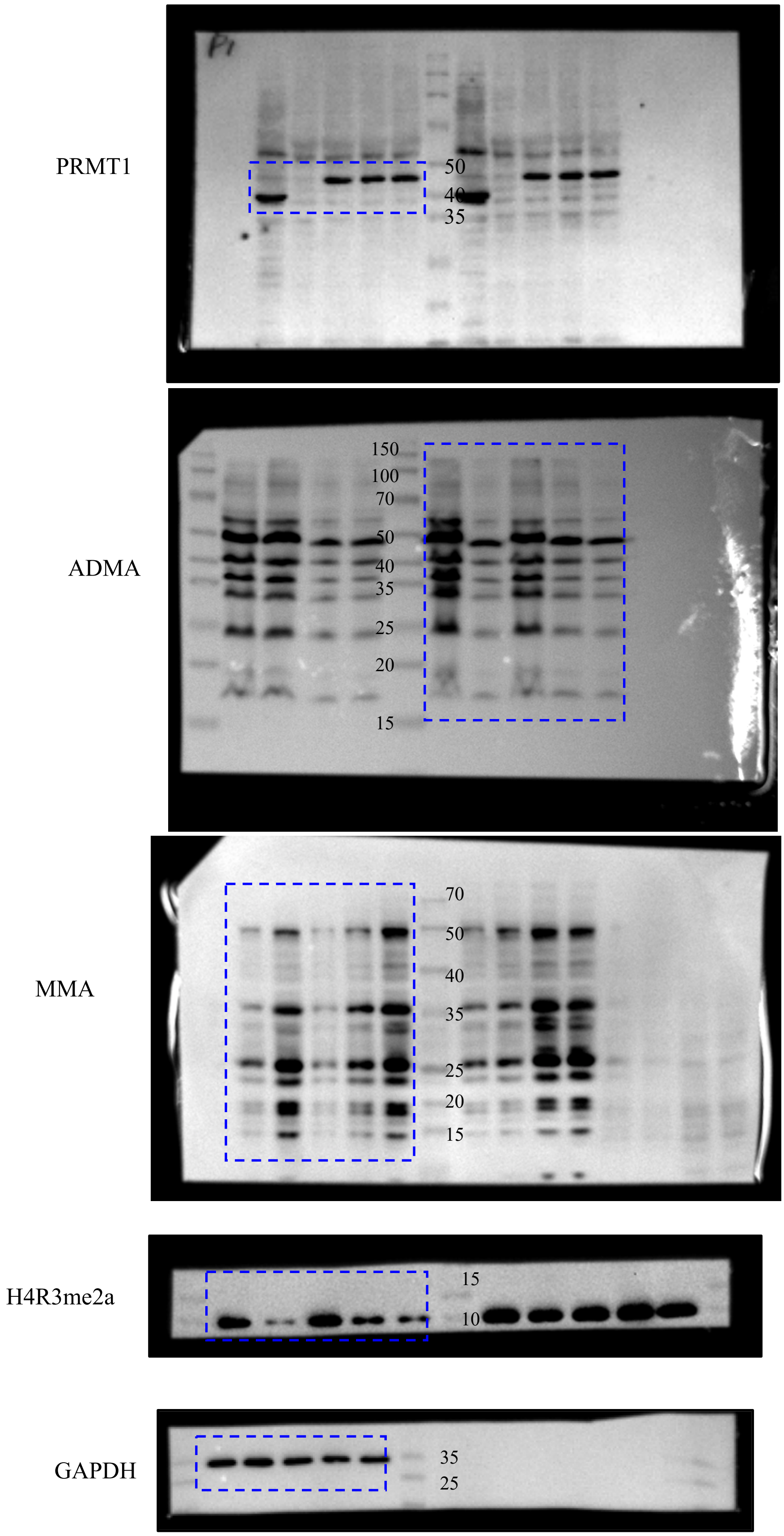
PRMT1
50
40
35
ADMA
150
100
70
50
40
35
25
20
15
MMA
70
50
40
35
25
20
15
H4R3me2a
15
10
GAPDH
35
25

Fig.S7F

ADMA

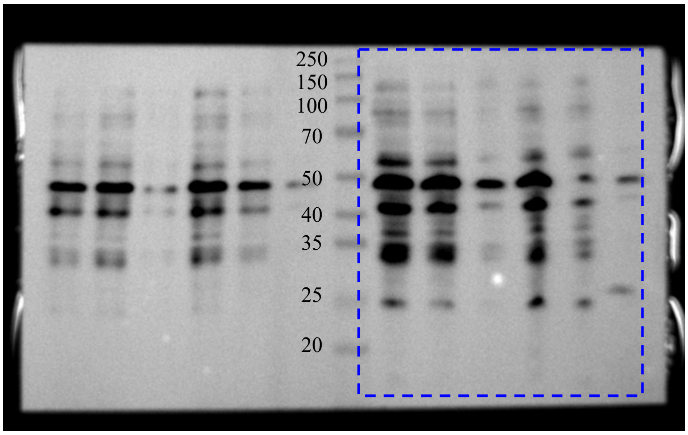

MMA

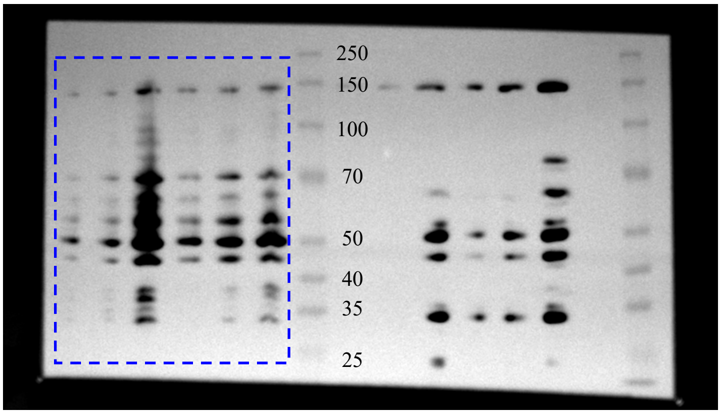

GAPDH

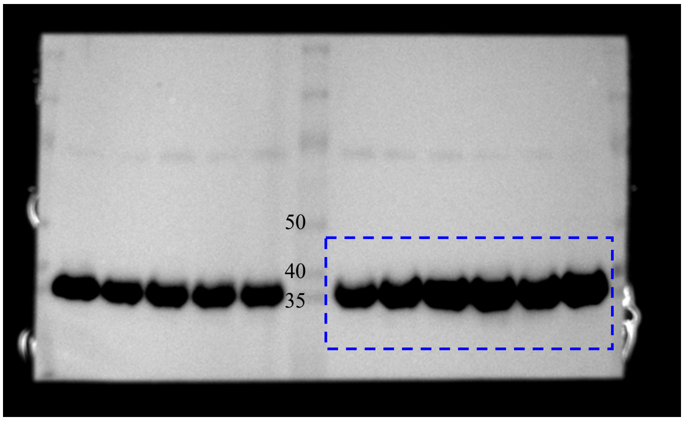

Supplement: Supplementary file 1 [file LSA-2025-03202_SdataF4_F5_F6_FS1_FS3_FS4_FS5_FS6_FS7.pdf]
