# Supplementary material for: PRMT1 oligomerization regulates RNA-binding protein cascade to promote pancreatic cancer
Source: Life Sci Alliance. 2025 Jul 17;8(9):e202503202. doi: 10.26508/lsa.202503202 (PMC12272085; doi:10.26508/lsa.202503202)
Supplement: Supplementary file 3 [file LSA-2025-03202_Supplemental_Data_1.docx]

Supplemental Data for

**PRMT1 Oligomerization Regulates RNA-Binding Protein Cascade to Promote Pancreatic Cancer**

Yanxia Ru^1,6^, Xinyi Zhou^2,6^, Xijiao Wang^2,6^, Wenxin Sun^2,6^, Yaohui He^2^, Guosheng Hu^2^, Wenjuan Li^2^, Die Hu^2^, Meizhi Jiang^2^, Zhiming Su^1^, Fengfeng Niu^1^, Gang Chen^4^, Jinzhang Zeng^2^, Sen-Fang Sui^1,5*^, Wen Liu^2*^, Yaowang Li^1*^, Siming Chen^2,3,7*^

***Correspondence:**

Siming Chen, simingchen@xmu.edu.cn

Yaowang Li, liyw@sustech.edu.cn

Wen Liu, w2liu@xmu.edu.cn

Sen-Fang Sui, suisf@mail.tsinghua.edu.cn

**This PDF file includes:**

**Figs. S1 to S8**

**KEY RESOURCES TABLE**

| REAGENT or RESOURCE | SOURCE | IDENTIFIER |
| --- | --- | --- |
| **Antibodies** |  |  |
| Myc-Tag (19C2) mAb | Abmart | Cat No. M20002S |
| Strep-Tag II Antibody | Abmart | Cat No. M40014S |
| Histone H4R3me2a (asymmetric) (pAb) | Active Motif | Cat No. 39006 |
| pan-Mono-Methyl Arginine Motif Rabbit pAb | Abclonal | Cat No. A17984 |
| Histone H4 Antibody | Cell Signaling Technology | Cat No. 2592S |
| Asymmetric Di-Methyl Arginine Motif [adme-R] Rabbit mAb | Cell Signaling Technology | Cat No. 13522S |
| PRMT1 Polyclonal antibody | Proteintech | Cat No. 11279-1-AP |
| Monoclonal ANTI-FLAG antibody | Sigma-Aldrich | Cat No. F1804 |
| GAPDH Monoclonal Antibody | Invitrogen | Cat No. MA1-16757 |
| **Gel filter column** |  |  |
| Superose6^TM^ Increase 10/300GL | Cytiva | Cat No. 29091596 |
| Superdex^TM^ 200 Increase 10/300GL | Cytiva | Cat No. 28990944 |
| **Recombinant DNA** |  |  |
| pET-28a-SUMO-StrepI-Tev-PRMT1 (32-371) | This Paper | N/A |
| pET-28a-SUMO-StrepI-Tev-PRMT1 (32-371) Y280A/H296A/T327A | This Paper | N/A |
| pET-28a-SUMO-StrepI-Tev-PRMT1 (32-371) W215A/Y220A/F222A | This Paper | N/A |
| pET-28a-SUMO-StrepI-Tev-PRMT1 (32-371) Y280A | This Paper | N/A |
| pET-28a-SUMO-StrepI-Tev-PRMT1 (32-371) H296A | This Paper | N/A |
| pET-28a-SUMO-StrepI-Tev-PRMT1(32-371) F222A | This Paper | N/A |
| pET-28a-SUMO-hnRNPA1 (1-320)-Flag | This Paper | N/A |
| pET-28a-SUMO-hnRNPA1 (186-320)-Flag | This Paper | N/A |
| pET-28a-SUMO-hnRNPA1 (1-196)-Flag | This Paper | N/A |
| pGEX-4T1-Tev-Fibrillarin (1-82) | This Paper | N/A |
| pMAL-C2X-hnRNPA2 (1-341)-Flag | This Paper | N/A |
| pGEX-4T1-Tev-FUS (1-526) | This Paper | N/A |
| pGEX-4T1-Tev-GAR (1-271) | This Paper | N/A |
| pGEX-4T1-Tev-hnRNPK (1-463) | This Paper | N/A |
| pGEX-4T1-Tev-Mettl14 (400-456) | This Paper | N/A |
| pMAL-C2X-Fibrillarin (1-321)-Flag | This Paper | N/A |
| pLKO.1-hygro-shPRMT1#1 | This Paper | N/A |
| pLKO.1-hygro-shPRMT1#2 | This Paper | N/A |
| pLKO.1-hygro-shNC | This Paper | N/A |
| pCDH-EF1-3 × Flag-PRMT1 | This Paper | N/A |
| pCDH-EF1-3 × Flag-PRMT1 (Y280A/H296A/T327A) | This Paper | N/A |
| pCDH-EF1-3 × Flag-PRMT (W215A/Y220A/F222A) | This Paper | N/A |
| pCDH-EF1-Myc-PRMT1 | This Paper | N/A |
| pCDH-EF1-Myc-PRMT1  (Y280A/H296A/T327A) | This Paper | N/A |
| pCDH-EF1-Myc-PRMT (W215A/Y220A/F222A) | This Paper | N/A |
| pCDH-EF1-3 × Flag-GAR1 (1-271) | This Paper | N/A |
| pCDH-EF1-3 × Flag-Fibrillarin (1-321) | This Paper | N/A |
| pCDH-EF1-3 × Flag-RBFOX2 (1-390) | This Paper | N/A |
| pCDH-EF1-3 × Flag-FUS (1-526) | This Paper | N/A |
| pCDH-EF1-3 × Flag-TAF15 (1-592) | This Paper | N/A |
| pCDH-EF1-3 × Flag-Nucleolin (1-710) | This Paper | N/A |
| pCDH-EF1-3 × Flag-hnRNPA1 (1-320) | This Paper | N/A |
| pCDH-EF1-3 × Flag-hnRNNPK (1-463) | This Paper | N/A |
| lentiCRISPR v2-sgPRMT1 | This Paper | N/A |
| lentiCRISPR v2 | Addgene | Cat No. 52961 |
| psPAX2 | Addgene | Cat No. 12260 |
| pCMV-VSV-G | Addgene | Cat No. 8454 |
| **REAGENT or RESOURCE** |  |  |
| Polyethylenimine Linear (PEI) MW25000 | YEASEN | Cat No. 40815ES03 |
| Thyroglobulin | Bioss | Cat No. bs-0291P |
| Strep-Tactin®XT 4Flow® high capacity resin | IBA Lifesciences | Cat No. 2-5030-010 |
| Pierce^TM^ Glutathione Agarose | Thermo Scientific™ | Cat No. 16101 |
| FLAG peptides | Sigma | Cat No. F3290 |
| AG RNAex Pro Reagent | Accurate Biotechnology | Cat No. AG21101 |
| Anti-DYKDDDDK G1 Affinity Resin | GenScript | Cat No. L00432-5 |
| Penicillin/Streptomycin | Yeasen | Cat No. 60162ES76 |
| Puromycin (Solution 10 mg/mL) | Yeasen | Cat No.60209ES10 |
| Hygromycin B | MCE | Cat No. HY-B0490 |
| Clarity Western ECL Substrate, 500 mL | Bio-Rad | Cat No.1705061 |
| Cell Counting Kit-8 | YEASEN | Cat No.40203ES76 |
| PrimeSTAR® GXL DNA Polymerase | TaKaRa | Cat No. R050A |
| Fetal bovine serum (FBS) | AMOBIO | Cat No. FBSAB001 |
| Dulbecco’s Modified Eagle Medium | Pricella | Cat No. PM150210 |
| (RPMI-1640) | Pricella | Cat No. PM150110 |
| **Deposited data** |  |  |
| RNA-seq | This Paper | PRJNA1230539 |
| Mass spectrometry | This Paper | PXD061743 |
| **Bacterial Isolates** |  |  |
| DH5α *E.coli* competent cells | Weidi | Cat. No. DL1004S |
| Rosetta2 (DE3) *E.coli* competent cells | Weidi | Cat. No. EC1014S |
| BL21 (DE3) competent cells | Weidi | Cat. No. EC1002S |
| **Experimental Models: Cell Lines** |  |  |
| Hela | ATCC | Cat. No. CCL-2 |
| HEK-293T | ATCC | Cat. No. CRL-3216, |
| PANC-1 | Servicebio | Cat. No. STCC11102P |
| MIA PaCa-2 | Servicebio | Cat. No. STCC11101P |
| **Oligonucleotides** |  |  |
| shPRMT1#1: GTGTTCCAGTATCTCTGATTA | Sangon | N/A |
| shPRMT1#2: CCGGCAGTACAAAGACTACAA | Sangon | N/A |
| sgPRMT1:  CGAGGCCGCGAACTGCATCA | Sangon | N/A |

**RESOURCE AVAILABILITY**

**Lead contact**

All further inquiries and requests for resources and reagents should be addressed to the Lead Contact, Siming Chen (simingchen@xmu.edu.cn)

**Materials availability**

The materials generated in this study are available under a Material Transfer Agreement.
